# Feasibility of delivering a randomized controlled trial of weighted blanket intervention to help agitation and disturbed sleep after brain injury

**DOI:** 10.3389/frsle.2024.1325175

**Published:** 2024-01-24

**Authors:** Laura Edwards

**Affiliations:** ^1^Division of Rehabilitation Medicine, University Hospitals of Derby and Burton NHS Foundation Trust, Derby, United Kingdom; ^2^Centre for Rehabilitation and Ageing Research, University of Nottingham, Nottingham, United Kingdom

**Keywords:** weighted blanket, sleep, acquired brain injury, actigraphy, neurorehabilitation, agitation

## Abstract

Sleep disturbance and agitation are common after acquired brain injury (ABI). Weighted blankets may help these symptoms in other conditions, but have not been trialed in ABI. We aimed to determine acceptability and feasibility of weighted blankets to aid sleep and agitation after ABI. We recruited participants into a non-blinded, randomized controlled study in an NHS inpatient neurorehabilitation unit. Participants were randomized 1:1 to weighted blanket or standard hospital linen. Participants underwent observation of sleep (including actigraphy) and behavior over 11 nights. Of 10 participants randomized, seven participants completed the study. Only 1 participant tolerated the weighted blanket throughout and only three wore the actigraph for all nights of the study. Participants found the weighted blanket hot, heavy and uncomfortable. The study was terminated early due to poor tolerance. Delivery of a definitive trial in this format would not be feasible.

## 1 Introduction

Acquired brain injury (ABI) is common and can be devastating, often affecting physical, cognitive, emotional, behavioral and social functioning. The most common cause of ABI is traumatic brain injury, commonly due to a fall or road traffic accident. After a severe traumatic brain injury, patients frequently experience agitation (Janzen et al., 2014), with a period of post traumatic amnesia, when they are disorientated, confused and unable to process and retain information. This may present as pacing, wandering and distress, and can be unpleasant for the patient, their friends, family and carers (Marshman et al., 2013). Sleep disturbance is also commonly seen after ABI (Wolfe et al., 2018). It is estimated that over two-thirds of patients on rehabilitation units have disturbed sleep (Barshikar and Bell, 2017) and traumatic brain injury patients in particular have been shown to struggle with insomnia, poor sleep efficiency and reduced sleep duration with frequent and earlier wakening (Mathias and Alvaro, 2012; Grima et al., 2016).

Treating these symptoms is challenging—it can be very difficult to reassure and orient someone who is unable to recall what they have been told. Medication can be used but may be sedating or predispose to other problem such as falls, seizures or cardiac problems (Mehta et al., 2018). One-to-one nursing (“specialling”) may be needed to maintain safety, but this can be perceived as intrusive [constantly being supervised and observed by another person (Moyle et al., 2011)], awkward and even threatening.

A weighted blanket is one which has been manufactured to be heavier than traditional bed linen (recommendations are for these to be around 10% of the weight of the person using it). This is suggested to provide “deep touch pressure sensory input”, which has been shown to reduce anxiety (Chen et al., 2013) and promote sleep (Ackerley and Olausson, 2015). It is suggested that the use of weighted blankets may avoid the use of sedating drugs and provide safe and calming (Mullen et al., 2008), non-restraining proprioceptive input, comfort and reassurance (Ackerley and Olausson, 2015).

The main research area for deep touch pressure originally was in children with developmental disorders such as autism and attention deficit hyperactivity disorder, with benefits shown in calming (Bestbier and Williams, 2017) and improved attention (Fertel-Daly et al., 2001; VandenBerg, 2001; Lin et al., 2014; Lee and Song, 2015) and reduced sleep latency (Hvolby and Bilenberg, 2011), although data on effectiveness are inconclusive (Hodgetts et al., 2011; Davis et al., 2013; Gringras et al., 2014).

Studies in psychiatric disorders and dementia have shown good levels of safety, tolerability and satisfaction of weighted blankets, with reduced fatigue, depression and anxiety (Ekholm et al., 2020; Harris and Titler, 2022). In a nursing-home-based study based, improvements were seen in sleep latency and night wakings in people using weighted blankets (Hjort Telhede et al., 2022). In chronic pain, the use of heavy weighted blankets was associated with reductions in pain perception but not in pain intensity, anxiety or sleep (Baumgartner et al., 2022).

A systematic review from 2020 across a range of populations concluded that weighted blankets could have some role in anxiety reduction but more research was needed into other areas, including insomnia (Eron et al., 2020).

To date, there has not been research into the use of weighted blankets in patients with ABI. If findings from other populations were replicated they could reduce distress and agitation and improve sleep quality. This, in turn, could contribute to reduced sedation and need for intensive nursing. It is not clear, though, that people with ABI will tolerate weighted blankets, given the frequency of agitation and the severity of sleep-wake cycle disturbance. It is not also not clear which outcome measures might best capture any impact from weighted blankets.

Against this background we designed and conducted a feasibility randomized controlled trial of weighted blankets in patients with ABI.

### 1.1 Aim

To determine the acceptability and feasibility of using weighted blankets to improve sleep and reduce agitation after ABI for patients undergoing inpatient neurorehabilitation.

## 2 Methods

This single-site unblinded randomized controlled feasibility study was sponsored and funded by University Hospitals of Derby and Burton NHS (National Health Service) Foundation Trust (ref UHDB/2020/024) and approved by Leeds West Research Ethics Committee (ref: 20/YH/0278). It was conducted at King's Lodge Neurorehabilitation Unit, a 19-bedded rehabilitation unit located in Florence Nightingale Community Hospital in Derby, United Kingdom.

Participants were aged over 18 years and weighed more than 50 kg. They had to be able to remove the blanket independently and not have claustrophobia, pain which could be exacerbated by the blanket, open wounds, or respiratory disorders. Participants had to be capable of giving informed consent, or if appropriate, have an acceptable individual capable of giving assent on the subject's behalf.

Participants in the intervention arm were provided with a weighted blanket of <10% of the participant's body weight. Weighted blankets were CE-marked and purchased from SensoryDirect.com at weights of 4.5, 6, and 7 kg. Participants were initially offered the blanket closest to no more than 10% of their weight i.e., 45 kg−59.9 kg = 4.5 kg blanket; 60–69.9 kg = 6 kg blanket; 70+ kg = 7 kg blanket. After noting some issues with ability to tolerate blankets early in the study, the final two participants in the intervention arm were also offered the opportunity to use lighter blankets if they found the initial weight was too heavy or uncomfortable for them in the hopes of aiding compliance with the intervention.

Participants in the control arm used standard hospital linen (sheets/blankets) for the duration of the study.

### 2.1 Study timeline

Baseline: Informed consent/assent given; baseline data collected; randomized to intervention/control; provided with actigraph to be worn throughout study.

Nights 1–2: all participants used standard hospital linen.

Nights 3–9: participants in weighted blanket arm used weighted blanket; participants in control arm used standard hospital linen.

Nights 10–11: all participants used standard hospital linen.

### 2.2 Measures

- Feasibility of recruitment (measured by number of eligible candidates, recruitment rates and problems encountered), retention (measured by attrition rates, with reasons recorded), randomization, data collection.
- Sleep: measured by actigraphy [a valid tool for assessing sleep in patients with brain injury in patients with limb movement and some awareness (Zollman et al., 2010)] and paper sleep chart, which is completed regularly on our rehabilitation unit for patients with sleep problems.
- Agitated behavior—Agitated Behavior Scale (Bogner et al., 2000) completed at the end of every night by the nurse looking after the patient during the night shift.
- Level of supervision (standard hourly checks vs. distant supervision vs. 1:1 nursing).
- Sedative/hypnotic medication used.
- Other interventions.

### 2.3 Sample size

No sample size calculation was performed. Twelve participants (six in each arm) were judged sufficient to answer feasibility questions.

### 2.4 Data collection and analysis

Custom designed case report forms were used to collect demographic data, and record sleep charts, use of blankets, agitated behavior scale, details of any interventions for each night of the study, details of adverse events, study discontinuation etc.

Data from these were collated into a spreadsheet. Actigraph data were analyzed using MotionWare software from CamnTech. Actigraph data analysis used MotionWare software (CamnTech). Data were analyzed using GraphPad Prism.

## 3 Results

The study was conducted between May 2021 and May 2023, with recruitment periods limited to non-summer months (September to May).

**Figure 1:**
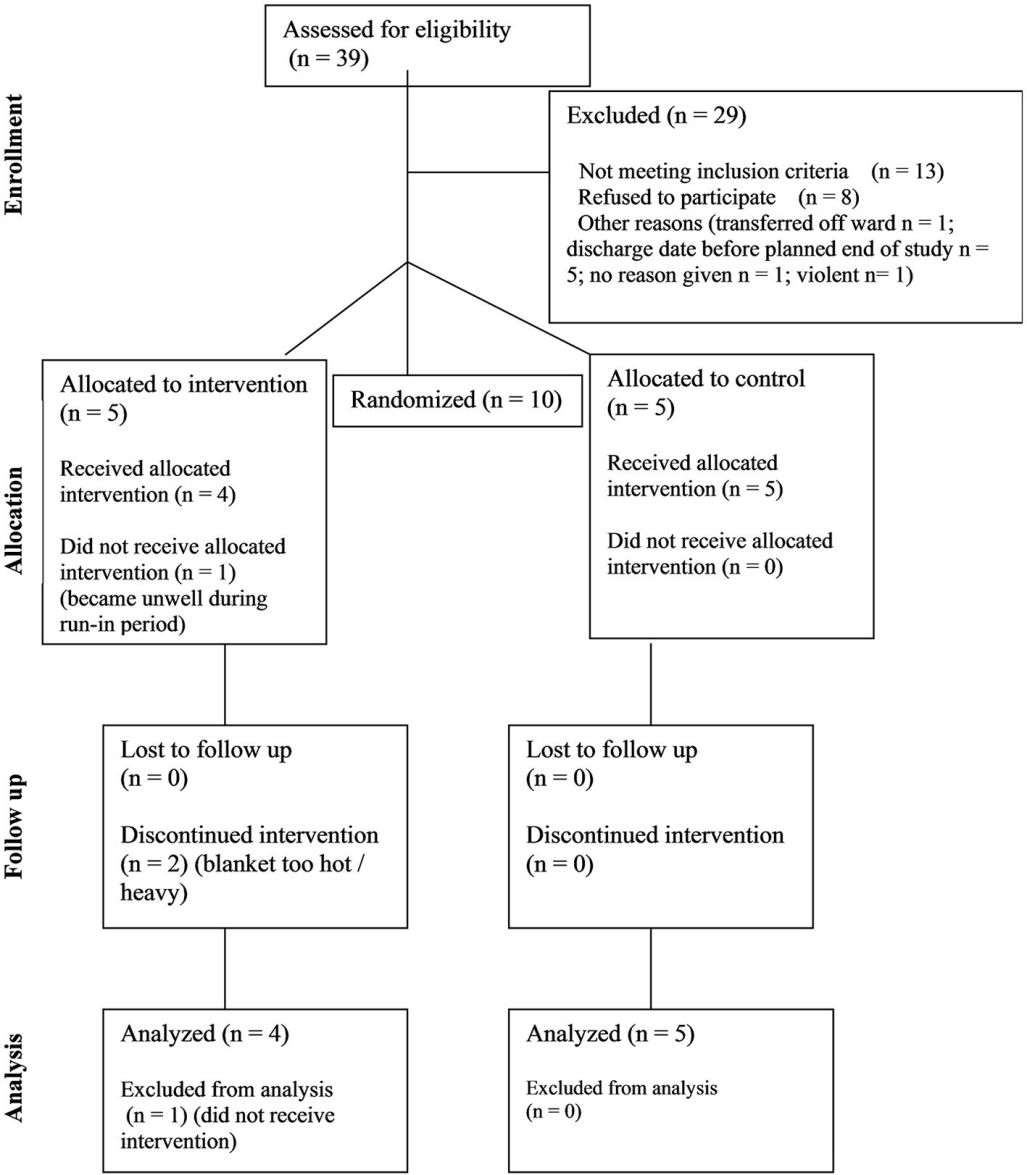
CONSORT diagram showing flow of participants through the study.

Thirty nine participants were assessed for eligibility. 29 were excluded. 10 participants were randomized; five to each arm. See Figure 1–CONSORT diagram.

There were no statistically significant differences between groups at baseline (age range 31–76; two females and three males in each group).

The most common diagnoses were traumatic brain injury (*n* = 4), then hypoxic brain injury (2), followed by infections (2), stroke (1) and metabolic brain injury (1).

Time since injury at time of recruitment ranged between 54 and 125 days (median 81 days) and time since admission to rehabilitation unit at time of recruitment ranged between 4 and 74 days (median 30.5 days). There was no statistically significant difference between time since injury or time on rehabilitation unit between the intervention and control groups.

### 3.1 Feasibility outcomes

#### 3.1.1 Recruitment

There were high rates of non-eligibility, commonly due to the presence of wounds (*n* = 3), respiratory problems (*n* = 3) or inability to remove the blanket due to weakness or executive dysfunction. Eight potential participants declined to participate.

#### 3.1.2 Retention

All participants in the control group completed all 11 nights in the study, whereas only one of the participants in the intervention group completed all 11 nights (Fisher's exact test *p* = 0.048). Participants in the intervention group spent a median of six nights (Interquartile Range, IQR 7.5) in the study and a median of one night (IQR 5.8) out of seven with a weighted blanket. In the intervention group one participant developed an intercurrent illness; two participants found the weighted blanket too hot/heavy/uncomfortable after the first night with weighted blanket (night 3 of the study) and one asked to be withdrawn from the study at night 10 (i.e., after all nights with weighted blanket).

#### 3.1.3 Randomization

No problems emerged around randomization.

#### 3.1.4 Recording of outcome measures

In total, the 10 participants spent 85 nights in the study. Sleep charts were completed for 58/85 (68%) nights. Actigraphs were worn for 67/85 (79%) nights. Actigraphs were worn for a median of three nights (IQR 8) in the intervention group and 10 (6.5) in the control group. Agitated behavior scale was completed for 43/85 (51%) nights. Medication and behavioral interventions data were collected for all participants at all time points. See Table 1 for details.

**Table 1:** Timeline of study for each participant showing completion of outcome measures.

**Night**		**1**	**2**	**3**	**4**	**5**	**6**	**7**	**8**	**9**	**10**	**11**
P01	Sleep chart											
	ABS											
	Actigraphy											
P02	Sleep chart											
	ABS											
	Actigraphy											
P03	Sleep chart											
	ABS											
	Actigraphy											
P04	Sleep chart											
	ABS											
	Actigraphy											
P05	Sleep chart											
	ABS											
	Actigraphy											
P06	Sleep chart											
	ABS											
	Actigraphy											
P07	Sleep chart											
	ABS											
	Actigraphy											
P08	Sleep chart											
	ABS											
	Actigraphy											
P09	Sleep chart											
	ABS											
	Actigraphy											
P10	Sleep chart											
	ABS											
	Actigraphy											

Green, complete for night in question; Amber, partially complete but insufficient for analysis; Red, not complete for night in question; Black, participant discontinued study.

P denotes participant identification.

ABS, agitated behavior scale.

### 3.2 Study termination

After the 10th participant (5th in intervention arm) was unable to tolerate the weighted blanket, it was decided to terminate the study rather than recruit the final 2 planned participants as it was felt that this would not add further information.

### 3.3 Clinical outcomes

No significant differences were seen between time points or groups when comparing sleep duration, waking episodes, Agitated Behavior Scale data, medication use or supervision level from paper records or actigrams.

### 3.4 Agreement between sleep charts and actigraphy

Hours of sleep and number of waking bouts were statistically significantly over- and under-reported respectively by paper sleep charts compared to actigrams (hours of sleep by paper chart median 8 h vs. actigram 6.55 h; Wilcoxon matched pairs signed ranks test *p* < 0.0001; number of waking bouts by paper chart median 1 vs. actigram median 41; Wilcoxon *p* < 0.0001).

## 4 Discussion

To our knowledge, this is the first study investigating the use of weighted blankets in hospital inpatients with ABI. It faced significant challenges around participant recruitment, retention and data collection and was terminated early.

Only 1 participant was able to tolerate the weighted blanket for all nights. The most common reason for discontinuation was finding the weighted blanket hot, heavy and uncomfortable. Only 5 participants wore their actigraphs for their whole time in the study.

Rates of tolerance and satisfaction with weighted blankets have varied across different studies. A large observational study in Sweden included over 4,000 mainly community-dwelling people who had been prescribed a weighted blanket for sleep problems, with a wide range of demographics (e.g., age range 1–103 years; most common diagnoses attention deficit hyperactivity disorder, anxiety and depression). Less than 50% of individuals continued to use the blanket over the follow up period (around 2 years) but reasons for discontinuation were not provided (Odeus et al., 2023). A nursing-home based study, also in Sweden, of 110 older people using weighted blankets at around 10% body weight showed that 38 (35%) participants found the blankets uncomfortable (Hjort Telhede et al., 2022). A study based in an American trauma unit showed that 11/12 participants who had been allocated to use a weighted blanket for five nights found the blanket either comfortable or “neutral” with only 1 participants describing it as uncomfortable, but a “common” complaint “*was that the blanket was too warm which was intensified by their already warm room*” (Warner et al., 2023). NHS hospital wards are recognized to be warm environments. For this reason, we elected not to recruit during particularly hot summer months, but restrict recruitment and study delivery to autumn, winter and spring. Nevertheless, participants reported feeling hot under the weighted blankets in our study. A suitable thermal environment is recognized as being key for sleep (Okamoto-Mizuno and Mizuno, 2012), and the bedding “microclimate” is particularly likely to disrupt sleep if it leads to an individual feeling too hot (Troynikov et al., 2018). NHS hospital wards are generally kept at warmer temperatures than bedrooms in the community [recommendations from The Sleep Council and The Sleep Charity are that ideal bedroom temperature is between 16 and 18C. Average room temperatures in United Kingdom (UK) homes are around 19C (Hulme et al., 2013); temperatures are not routinely measured on our rehabilitation unit, but another unit in the UK recorded average night time temperatures in patient rooms between 21.7 and 25.3 C (Yelden et al., 2015), and previous work has shown that NHS hospitals frequently exceed temperatures of 24 C overnight (Lomas and Giridharan, 2012; Giridharan et al., 2013)] so a higher ambient temperature with the addition of thicker bedding could well be an important contributory factor to the difficulty tolerating the blankets in this study, and also in comparison to other studies conducted in care homes or individuals' own homes which may have been a few degrees cooler. It is possible that the ambient conditions in UK NHS settings are different from those seen in other countries with more luxurious healthcare estates which are likely to allow more zoning of climate controlled (the unit participating in this study is centrally heated in winter, but not air conditioned, as is the case for much of the NHS estate).

Potential participants who had pain which could be exacerbated by weighted blankets were not included in this study. Nevertheless, the most common reason for discontinuation was finding the blankets hot, heavy and uncomfortable and it is also noteworthy that wearing actigraphs was not tolerated by many of the participants in the study—only five participants wore their actigraphs for their whole time in the study. A recent systematic review demonstrated high levels of sensory sensitivity, including tactile sensitivity, after brain injury (albeit with a focus on light and sound sensitivity) (Thielen et al., 2023), and there is also evidence that brain injury may induce allodynia, where a normally non-painful stimulus can induce pain (Irvine et al., 2018). Providing extra sensory input, through the actigraph +/- blanket, may have been overwhelming to participants who were already adjusting to new levels of sensory sensitivity, as our participants were still in a relatively early period of ABI recovery, with a median period of <3 months since the injury, whereas recovery can continue for several years, particularly with rehabilitation input (Wilson et al., 2017; Knox and Douglas, 2018; Williams et al., 2020). It is therefore possible that introducing the weighted blanket at a later stage of rehabilitation could have given different outcomes.

Outcome measures were frequently not completed. Staff do perform hourly rounding overnight but adding in (yet more) paperwork (Cooper et al., 2021) for completion in a busy clinical environment may understandably have been less of a priority, particularly at the end of a night shift when fatigue and sustained attention are reduced (James et al., 2021). For this feasibility study, we did not have inclusion criteria around sleep symptomatology. As discussed above, the role of weighted blankets in insomnia management remains debated (Gringras et al., 2014; Eron et al., 2020), with suggested benefits perhaps more related to comfort, reduced anxiety, and time spent resting in bed. It is possible that participants who identified having poorer sleep may have been less likely to withdraw from the study but this can only be speculative. Our experience is that there may not be consistent correlation between objective and subjective sleep measures following ABI, as has been reported elsewhere (Lan Chun Yang et al., 2021). We included participants with physical, cognitive and behavioral consequences of ABI, as these are commonly seen together (Pasquina et al., 2014). It is possible that restricting inclusion criteria to individuals with more specific impairments and symptoms could have resulted in different outcomes, but this would also likely have further restricted recruitment.

Wrist-worn actigraphs were much more sensitive at detecting sleep/wake episodes and duration of sleep than hourly observations, which is in keeping with findings from another recent study of sleep after ABI which showed over-estimation of sleep by sleep logs (Weppner et al., 2023). Actigraphy is less invasive than full polysomnography and hopefully more easily tolerated, and has been successfully used in other sleep and ABI studies (El-Khatib et al., 2019; Weppner et al., 2023). However, in our study, several participants disliked wearing the wrist actigraph and so removed it. Furthermore, several participants in this study were relatively immobile, spending a lot of time in bed or in a wheelchair and so using movement as a proxy for sleep was arguably less reliable than it might be in a more active population. Recent evidence suggests that actigraphy is a poor substitute for polysomnography in patients with brain injury, and frequently underestimates sleep disruption (Zeitzer et al., 2020).

Overall, this study protocol was not feasible to roll out to a larger scale study to assess the use of weighted blankets for agitation and disturbed sleep after ABI. It is helpful to demonstrate some of the challenges experienced in conducting this type of research, and to emphasize the importance of feasibility and pilot studies.

## Data availability statement

The raw data supporting the conclusions of this article will be made available by the authors, without undue reservation.

## Ethics statement

The studies involving humans were approved by Leeds West Research Ethics Committee (ref: 20/YH/0278). The studies were conducted in accordance with the local legislation and institutional requirements. Written informed consent for participation in this study was provided by the participants or by participants' consultee.

## Author contributions

LE: Conceptualization, Formal analysis, Funding acquisition, Investigation, Methodology, Project administration, Writing – original draft, Writing – review & editing.
